# The Complexity Behavior of Big and Small Trading Orders in the Chinese Stock Market

**DOI:** 10.3390/e25010102

**Published:** 2023-01-04

**Authors:** Yu Zhu, Wen Fang

**Affiliations:** Department of Finance, School of Economics and Management, Beijing Jiaotong University, Beijing 100044, China

**Keywords:** trading order, complexity behavior, volume, microstructure, Chinese market

## Abstract

The Chinese stock market exhibits many characteristics that deviate from the efficient market hypothesis and the trading volume contains a great deal of complexity information that the price cannot reflect. Do small or big orders drive trading volume? We studied the complex behavior of different orders from a microstructure perspective. We used ETF data of the CSI300, SSE50, and CSI500 indices and divided transactions into big and small orders. A multifractal detrended fluctuation analysis (MFDFA) method was used to study persistence. It was found that the persistence of small orders was stronger than that of big orders, which was caused by correlation with time. A multiscale composite complexity synchronization (MCCS) method was used to study the synchronization of orders and total volume. It was found that small orders drove selling-out transactions in the CSI300 market and that big orders drove selling-out transactions in the CSI500 market. Our findings are useful for understanding the microstructure of the trading volume in the Chinese market.

## 1. Introduction

Many transactions take place in the stock market every day and these transactions cause market fluctuations. The Chinese stock market has many characteristics that deviate from an efficient market [1,2] and financial anomalies, such as momentum and reversal effects in the stock market, show that price does not fully reflect market information. The trading volume contains a great deal of complexity information that price cannot reflect as a direct description of stock trading. The stock trading volume contains a lot of information, including fundamental stock trading information [3] and long-term stock market performance data [4]. For stock technical indicators, the trading volume can increase information on price and earnings [5,6], reflect volatility information about the stock [7], and provide international earnings spillover information [8]. Many volume studies have linked volume to expected returns. The volume–price relationship is a key issue and is important in the stock market [9,10]; the correlation between them changes with time [11]. The relationship between volume and price volatility has also been studied as it affects the stock market [12], the money market [13] and the foreign exchange market [14]. Some researchers have suggested that risk exposure [15] and household belief dispersion [16] are related to the trading volume of stocks. The driving force behind the trading volume is a problem that has concerned many investigators [17,18]. Studies undertaken include those from the investor’s perspective and consider which types of investors are the main drivers of trading volume. With respect to trading volume, buying and selling-out volume have been investigated separately [19]. In the present study, we sought to examine the information contained in the volume microstructure. The financial market is similar to a physical system made up of numerous interacting agents and many economists have used physical methods to study its complexity [20]. To investigate the complex information contained in trading volume data, we applied multifractal and entropy methods.

The Peter fractal market hypothesis states that the stock price follows fractional Brownian motion and challenges the strict assumption of an efficient market [21]. Hurst found that the current value of the time series affects the future value in a way that transcends random perturbations [22]. The study defined this phenomenon as the long memory of the time series and proposed the use of rescaled range analysis (R/S) for measurement. Mandelbrot proposed fractional Brownian motion (FBM) in 1968, developing a model combined with the Hurst index to form a well-established research system [23]. Peng proposed the detrending fluctuation analysis method (DFA) applied to the investigation of DNA sequences [24]. This method is more accurate and easier to implement than the R/S method and has gradually become the mainstream method for measuring long-term memory. Kantelhardt proposed the multifractal detrended fluctuation analysis (MFDFA) method based on improvement of the detrended fluctuation analysis (DFA) method, extending the method to multiple scales to correspond more closely to the reality of financial data [25]. Economists have applied long memory to non-stationary time series in the financial market and used multifractal methods to study the stock market [26,27], the futures market [28], the foreign exchange market [29], and the Bitcoin market [30,31]. Thompson verified improved fitting using the MFDFA method when measuring financial time series generalized Hurst indices and multifractal spectra [32].

In 1948, Shannon proposed the concept of information entropy and suggested that the greater the entropy, the greater the uncertainty of the variable, and the greater the amount of information required. Many scholars have extended the entropy method and proposed new entropy measurement methods, such as Deng entropy [33], dispersion entropy [34], and multiscale entropy [35]. The application of entropy in finance is based on information entropy. In 1972, Philippatos and Wilson first applied entropy to finance, building portfolios with minimal entropy, and received considerable returns [36]. The concept of entropy is applied in options pricing [37], risk measurement [38], and utility calculations [39]. In the stock market, entropy is used in the study of volume heterogeneity [40], volatility forecasting [41,42], and the investigation of stock market regularity during turbulence periods [43]. In recent years, entropy has been used to measure the similarity between financial markets [44], to study the synchronization of stock returns [45], and to determine the similarity between stock and commodity markets [46]. Pincus proposed an approximate entropy method [47] to measure time series complexity. Richman proposed a more accurate sample entropy method based on the approximate entropy [48]. Costa introduced a coarse-graining procedure to assess multiscale entropy at multiple timescales [49]. Xu researched the complexity problem of time series. Their study combined the entropy measurement method with the complexity invariant distance (CID) and proposed a multiscale composite complexity synchronization (MCCS) approach, which can replace cross-sample entropy when calculating the similarity of different time series [50].

There have been many investigations regarding the microstructure of the stock market. The trading volume is often included in microstructure research as trading volume has a driving effect on volatility, opening price, and income spillover [5,7,8,51]. Suominen used a game model to study the information content of the trading volume [52], while Wang studied the circuit breakers and volume structure of the Chinese stock market [53]. Ormos studied the impact of the financial crisis on the microstructure of the trading volume [54]. Xu first investigated the microstructure of the Chinese stock market in terms of volume and volatility [55], while Covrig and Ng studied the driving forces behind trading volume from an investor perspective [18]. Alvarez-Ramírez and Rodríguez used the DFA method to study the temporal correlation of trading volume [56]. Lee observed a stronger cross-correlation in stock buy volume from a buy and sell trade perspective [19]. The previous studies only investigated the microstructure of trading volume; however, we have applied economic physics methods to investigate the complexity behavior of trading volume. For the benefit of the reader, Table 1 summarizes previous studies and highlights the contribution of this paper. The driving influences of volume for big and small orders are conceivably different, being affected by time correlation, and selling transactions are more likely to have a clear driving force. This is a hypothesis that we discuss in the paper.

We aim to study the complexity behavior of different orders to find which orders drive the trading volume of the Chinese stock market. We investigate the fractal characteristics of trading volume and the complexity synchronization between the total volume and trading orders. We use the ETF data of the CSI 300, the SSE 50, and the CSI 500 indices from the Chinese stock market. We divide transactions into buying transactions and selling-out transactions and discuss their complexity characteristics. The MFDFA method is used to obtain a multifractality index and the fractal spectrum of the volume. In addition, the fractal characteristics of different markets are compared. We also plot the multifractality curves and analyze the reasons for the fractal characteristics of the three markets using the shuffle sequence. We find that small orders are persistent. On this basis, we analyze the complexity synchronization of the orders and total volume. We find that the transactions are influenced by different orders in different markets. For the selling-out market, small orders are the leading force in CSI300, while big orders are the main force in CSI500. This feature is caused by the correlation of time series.

The paper is organized as follows: Section 2 describes the ETF data and order transaction indicators and provides detailed MFDFA and MCCS calculations. Section 3 provides a simple explanatory analysis of the data, presents descriptive statistics and discusses their characteristics. Section 4 discusses the fractal characteristics and complex synchronization characteristics of orders and discusses them in relation to the findings of earlier studies. Section 5 gives the concrete value of the investment, considers the transferability of the methodology, and suggests future research directions.

## 2. Data and Methodology

### 2.1. Data

ETF is the exchange-traded fund. ETF tracks the sector index or the market index and can be purchased and redeemed at trading time or traded in the market like stocks. Investors can use ETFs for arbitrage; in the Chinese market, ETFs cannot be short sold. This paper uses data from the CSI300 ETF, SSE50 ETF, and CSI500 ETF from 1 January 2018 to 30 May 2022. CSI300 is the most representative index of the Chinese stock market and reflects the overall situation of China’s A-share market. The SSE50 is composed of 50 stocks with the highest dividend yield and the largest cash dividend and reflects the performance of leading enterprises in the Chinese market. The CSI500 reflects the stock performance of growth companies in the Chinese market. We selected the largest of the three index ETFs: Huatai-PB CSI300 ETF (510300), ChinaAMC SSE50 ETF (510050), and ChinaSouthern CSI500 ETF (510500).

The data includes the daily total volume and the daily trading order volume; we select the initiative buying and selling-out volume. The initiative buying is to agree at the lowest price of the selling orders; the initiative selling-out is to agree at the highest price of the buying orders. For the total volume, we use the total trading shares of the buying and selling-out. For trading orders, we divide trading orders into small orders and big orders.Transactions with a turnover of less than 40,000 yuan represent small orders, and transactions with a turnover of more than 1 million yuan represent big orders. The data was obtained from the Wind financial terminal.

### 2.2. MFDFA Method

The MFDFA method divides time series into sub-intervals and eliminates local trends, fits a residual sequence function, calculates the generalized Hurst exponent, and expresses the multifractal characteristics of time series through the power law correlation of functions and multifractal spectra. The MFDFA method is briefly described below.

Given a sample time series x(t)(t=1,2…N), the sequence length is *N*. The MFDFA method consists of several steps:

*Step* *1* Construct a time series side, where $\bar{x}=\sum_{t=1}^{N}x\left(t\right)$
  (1) $$X\left(i\right)=\sum_{t=1}^{i}\left[x\left(t\right)-\bar{x}\right],i=1,2,\ldots ,N$$
*Step* *2* Split the sub-interval

We divide X(i) into $N_{s}=int\left(N/s\right)$ with *s* as the equal interval; the length of the time series *N* is not always an integer multiple of the interval *s*. We ensure that the information of the sequences is included in the segments, thereby, the sequence obtains $2N_{s}$ segments.

*Step* *3* Local trend elimination

For each segment υ, the trend can be eliminated with a local polynomial, where m=3 is selected

(2) $$\begin{array}{c} \gamma_{\upsilon }\left(i\right)=X\left[\left(\upsilon -1\right)s+i\right]-x_{\upsilon }\left(i\right) \end{array}$$

(3) $$\begin{array}{c} x_{\upsilon }\left(i\right)=\delta_{m}i^{m}+\ldots +\delta_{1}i+\delta_{0} \end{array}$$

(4) $$\begin{array}{c} F^{2}\left(s,\upsilon \right)=\frac{1}{s}\sum_{i=1}^{s}{\left[\gamma_{\upsilon }\left(i\right)\right]}^{2} \end{array}$$

*Step* *4* Calculates the volatility function, where *q* is not equal to 0
  (5) $$F_{q}\left(s\right)={\left\{\frac{1}{N_{S}}\sum_{\upsilon =1}^{N_{s}}{\left[F^{2}\left(s,\upsilon \right)\right]}^{\frac{q}{2}}\right\}}^{\frac{1}{q}}$$
*Step* *5* Calculates the scale variable

The power-law function $F_{q}\left(s\right)$ satisfies the following relationship $F_{q}\left(s\right)\propto s^{h(q)}$, h(q) is the slope of $\log F_{q}\left(s\right)=h\left(q\right)\log\left(s\right)+\log C$, called the generalized Hurst index, adjust *q* to get a different h(q). When q>0, the characteristics of large fluctuating segments are indicated, and when q<0, the characteristics of small fluctuating segments are indicated. When h(q)>0.5, the sequence is persistent and the previously ascending sequence still rises afterwards; When 0<h(q)<0.5, the sequence is anti-persistent and the previously ascending sequences revert to the mean. Then, we plot multifractal spectra to present the fractal behaviors of the sequences [57].

(6) $$\begin{array}{c} \tau (q)=qh(q)-1 \end{array}$$

(7) $$\begin{array}{c} \alpha \left(q\right)=h\left(q\right)+qh^{\prime }\left(q\right) \end{array}$$

(8) $$\begin{array}{c} f(\alpha )=q\alpha (q)-\tau (q) \end{array}$$

We use α to describe the time series’ variation and depict the partial multifractal characteristics. The smaller α, the greater the singularity. τ(q) is the Renyi index, f(α) is the multifractal spectrum. The greater the fractal spectrum width $\alpha_{\max}-\alpha_{\min}$, the more significant the fractal and the greater the slope of the Hurst index. $\Delta f\left(\alpha \right)=f{\left(\alpha \right)}_{\max}-f{\left(\alpha \right)}_{\min}$; the larger the Δf(α), the greater the difference between series, the greater the fluctuation, the more uneven the distribution.

### 2.3. MCCS Method

For financial time series, this method first follows a coarse-graining procedure to the series and then calculates a complexity synchronization measure, which is composed of sample entropy and complexity invariant distance.

Given two equal length time series $x_{n}$, $y_{n}$ with multiscale scale factor τ, where $N_{\tau }=int\left(n/\tau \right)$,

(9) $$\begin{array}{c} x_{j}^{\left(\tau \right)}=\frac{1}{\tau }\sum_{i=(j-1)\tau +1}^{j\tau }x\left(i\right),1\leq j\leq N_{\tau } \end{array}$$

(10) $$\begin{array}{c} y_{j}^{\left(\tau \right)}=\frac{1}{\tau }\sum_{i=(j-1)\tau +1}^{j\tau }y\left(i\right),1\leq j\leq N_{\tau } \end{array}$$

The sample entropy is an improvement on the approximate entropy and is calculated as follows: For the time series $x_{n}$, *m* is the giving dimension, which can constitute the m-dimensional vector $x^{m}\left(i\right)=\left\{x_{i},x_{i+1},\ldots ,x_{i+m-1}\right\}$, where i∈{1,2,…,n−m}; in this paper, m=2. The distance between two vectors $x^{m}\left(i\right)$ and $x^{m}\left(j\right)$ is $d\left(x^{m}\left(i\right),x^{m}\left(j\right)\right)=max\left(|x_{i+k}-x_{j+k}|:0\leq k\leq m-1\right)$; when the distance *d* is less than the tolerance level *r*, the vector $x^{m}\left(i\right)$ is said to be close to $x^{m}\left(j\right)$, $N_{i}^{m}\left(\hat{r}\right)$ is the number of vectors close to the vector $x^{m}\left(i\right)$, tolerance *r* is 0.15 times the standard deviation in this paper, and the probability of being close to $x^{m}\left(i\right)$ is

(11) $$C_{i}^{m}\left(\hat{r}\right)=\frac{N_{i}^{m}\left(\hat{r}\right)}{n-m-1}$$

The mean for this probability is

(12) $$C^{m}\left(\hat{r}\right)=\frac{1}{n-m}\sum_{i=1}^{n-m}C_{i}^{m}\left(\hat{r}\right)$$

The sample entropy is defined as [48]

(13) $$\mathrm{SampEn}\left(x,m,\hat{r}\right)=-\log\left(\frac{C^{m+1}\left(\hat{r}\right)}{C^{m}\left(\hat{r}\right)}\right)$$

The generalized complexity invariant distance measures the complexity relationship between two-time series, *x* and *y*, according to the Minkowski distance,

(14) $$\begin{array}{c} \Delta x=\{x_{2}-x_{1},x_{3}-x_{2},\ldots ,x_{n}-x_{n-1}\} \end{array}$$

(15) $$\begin{array}{c} \Delta y=\{y_{2}-y_{1},y_{3}-y_{2},\ldots ,y_{n}-y_{n-1}\} \end{array}$$

(16) $$\begin{array}{c} {∥x∥}^{p}=\sum_{i=1}^{n}{\left|x_{i}\right|}^{p} \end{array}$$

(17) $$\begin{array}{c} {∥y∥}^{p}=\sum_{i=1}^{n}{\left|y_{i}\right|}^{p} \end{array}$$

The generalized complexity invariant distance is calculated as

(18) $$\mathrm{GCID}\left(x,y,p\right)={∥x-y∥}^{p}\times \frac{\max\{∥\Delta x{∥}^{p},∥\Delta y{∥}^{p}\}}{\min\{∥\Delta x{∥}^{p},∥\Delta y{∥}^{p}\}}$$

We define $\mathrm{MCCS}\left(x,y,p,\tau \right)=\mathrm{CCS}\left(x^{\left(\tau \right)},y^{\left(\tau \right)},p\right)$, where CCS is calculated from the sample entropy and the generalized complexity invariant distance,

(19) $$\mathrm{CCS}\left(x,y,p\right)={\left[\mathrm{SampEn}(|x-y\right|}^{p},m,\hat{r}{)\times \mathrm{GCID}\left(x,y,p\right)]}^{\frac{1}{p}}$$

The correlation coefficient $\rho_{\mathrm{MCCS}}$ of multiscale complexity synchronization between *x* and *y* is defined as,

(20) $$\rho_{\mathrm{MCCS}}\left(x,y,p,\tau \right)=\exp\left\{-\mathrm{MCCS}\left(x,y,p,\tau \right)\right\}$$

## 3. Explanatory Analysis

Table 2 shows the order transaction of the CSI300, SSE50 and CSI500 markets. The SSE50 market has a higher volume and the CSI500 market has a lower volume. For different orders, the mean and standard deviation of buying and selling transactions for big orders are higher than those for small orders, which indicates that the big orders have a higher absolute volume than small orders and dramatic volatility. The volume of the three markets shows peak characteristics and the JB test results strongly reject the normal distribution hypothesis. This suggests that the volume of the three markets does not closely follow a normal distribution and needs to be studied from the perspective of a fractal market.

We consider the inconsistencies between the different markets; the data is normalized,

(21) $$x_{\mathrm{new}}=\frac{x-x_{\min}}{x_{\max}-x_{\min}}$$

**Figure 1:**
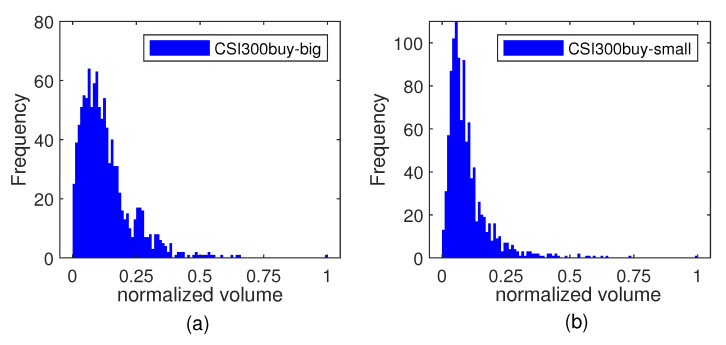

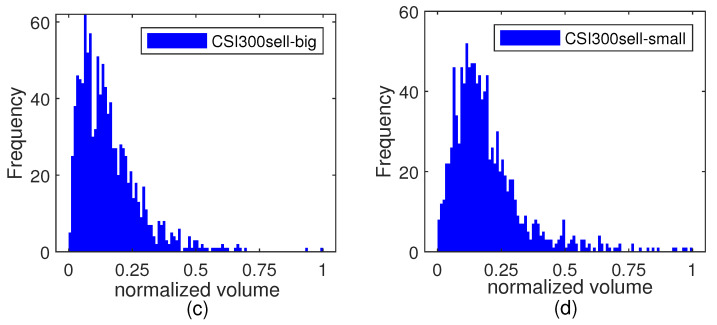
Distribution histogram of big and small orders in the CSI300 market. (**a**) big order of buying transaction. (**b**) small order of buying transaction. (**c**) big order of selling transaction. (**d**) small order of selling transaction.

**Figure 2:**
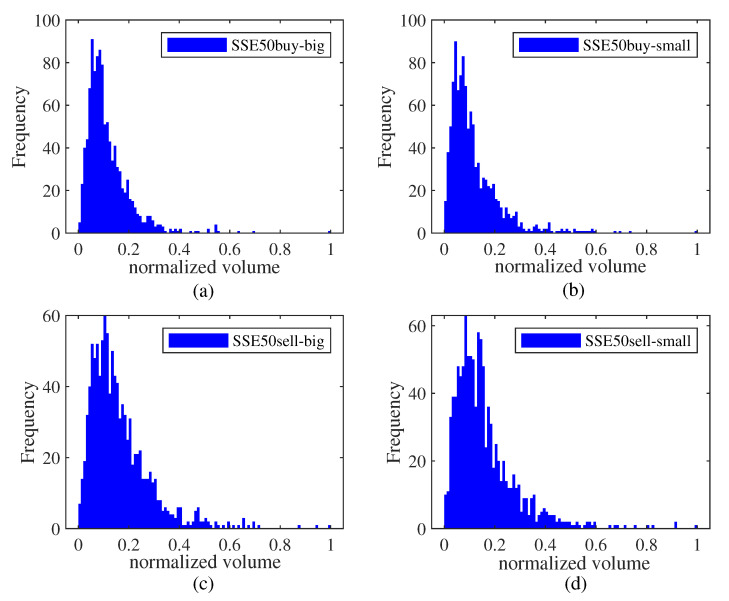
Distribution histogram of big and small orders in the SSE50 market. (**a**) big order of buying transaction. (**b**) small order of buying transaction. (**c**) big order of selling transaction. (**d**) small order of selling transaction.

**Figure 3:**
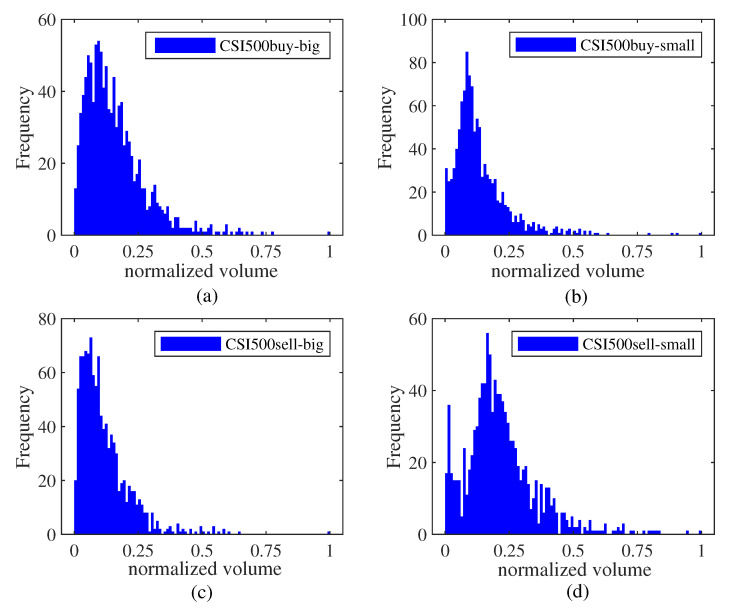
Distribution histogram of big and small orders in the CSI500 market. (**a**) big order of buying transaction. (**b**) small order of buying transaction. (**c**) big order of selling transaction. (**d**) small order of selling transaction.

To study the characteristic of big and small orders in different markets, Figure 1, Figure 2 and Figure 3 present relevant histograms. The big and small orders in the three markets have obvious spikes and right-bias characteristics.

## 4. Results and Discussion

### 4.1. Fractal Characteristics of Big and Small Orders

**Figure 4:**
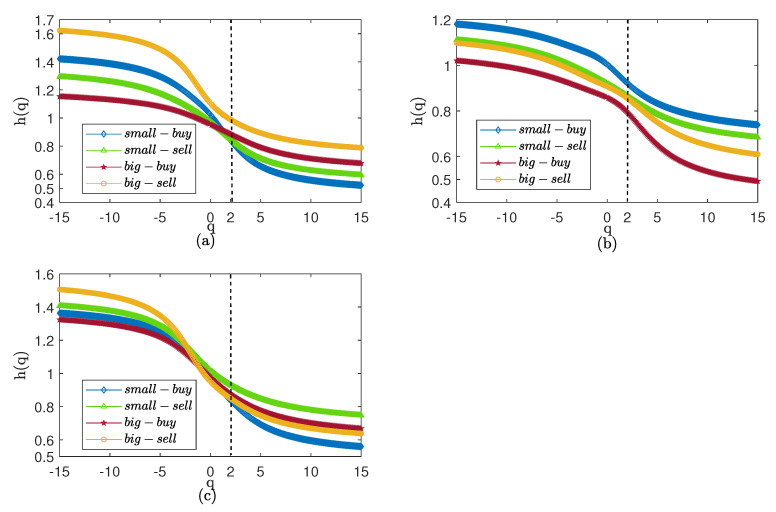
Hurst curve of small and big orders. (**a**) CSI300. (**b**) SSE50. (**c**) CSI500.

We divide big and small orders into buying and selling-out trades and obtain four types of orders. We plot the generalized Hurst index curve; the fluctuation scale *q* is selected as (−15,15). The result are presented in Figure 4. The Hurst curves of the three markets are all above 0.5, the transaction shows persistence, and the persistence of small fluctuations is especially significant. In the CSI300 market, the selling-out transaction for big orders has a stronger persistence. The buying transactions for big orders have lower persistence with small fluctuations, while the buying transactions for small orders have lower persistence with significant fluctuations. In the CSI500 market, the different orders have similar persistence and the persistence has differences including both minimal and great fluctuations. For the SSE50 market, the buying transactions of small orders show a high degree of persistence and the persistence of the big orders is lower than that of the small orders. When q>10, the Hurst index for big buying transactions is close to 0.5, indicating that, when significant fluctuations occur, the market volume is random and there is no obvious persistence or anti-persistence.

**Figure 5:**
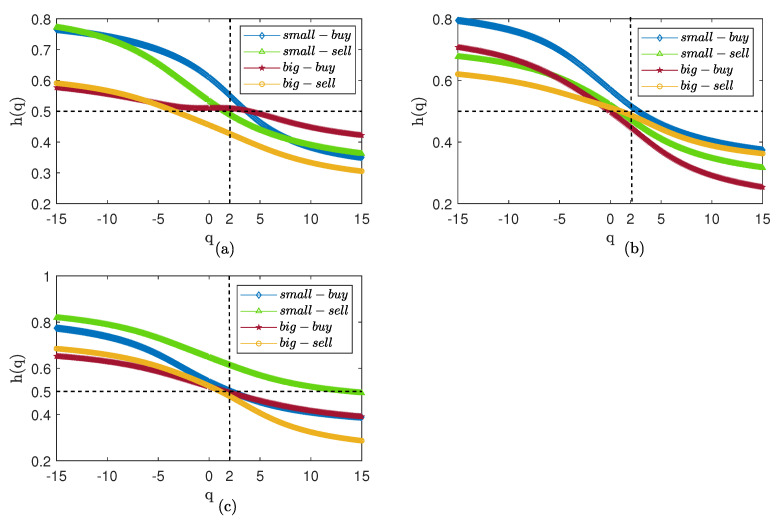
Hurst curve of small and big orders after shuffle. (**a**) CSI300. (**b**) SSE50. (**c**) CSI500.

The Hurst curve after the shuffle of the original sequence is presented in Figure 5. The Hurst index of the three markets significantly reduces, showing that the persistence of the transaction is caused by the time series correlation. For the CSI300 market, the small orders after the shuffle have apparent persistence under small fluctuations. The transaction of big orders is close to 0.5 when *q* is near 0, indicating that, when small fluctuations occur, the buying transactions for big orders have no prominent memory characteristics. The SSE50 market shows the characteristic of persistence with small fluctuations and anti-persistence with big fluctuations; the buying transactions for small orders is highly persistent. In the CSI500 market, the selling-out transaction of small orders always shows persistence, indicating that small orders are the force behind selling-out transactions.

**Figure 6:**
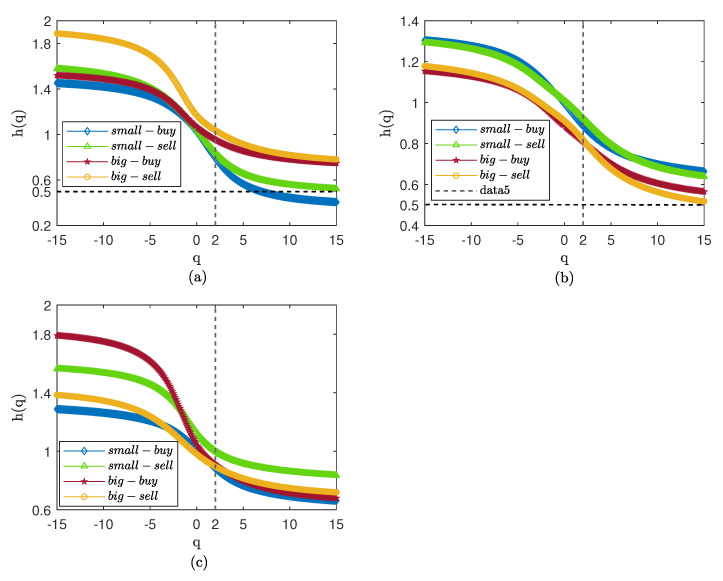
Hurst curve of small and big orders with weekly volume. (**a**) CSI300. (**b**) SSE50. (**c**) CSI500.

**Figure 7:**
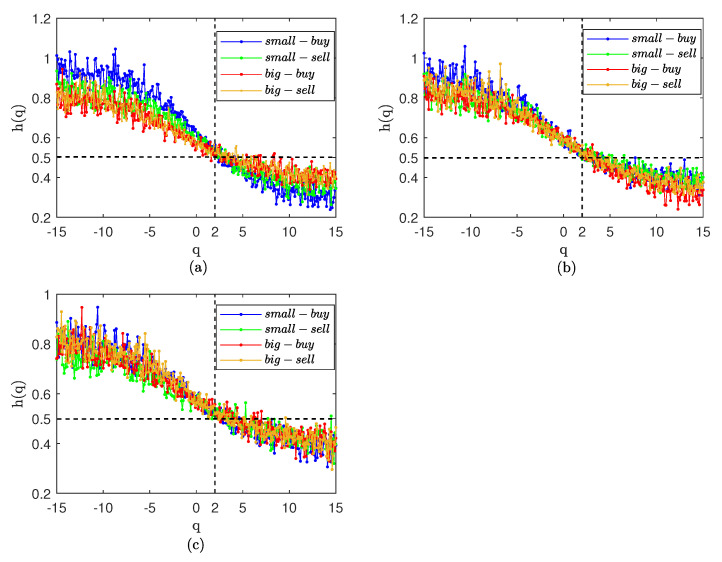
Hurst curve of small and big orders with weekly volume after shuffle. (**a**) CSI300. (**b**) SSE50. (**c**) CSI500.

We report multifractal results for weekly volume in Figure 6 and Figure 7. The results are similar to the daily volume in Figure 4, with strong persistence in all three markets. In contrast to Figure 5, the generalized Hurst curve in Figure 7 is not smooth and very close, but shows persistence with small fluctuations and anti-persistence with big fluctuations; the persistence of small orders is higher than that of big orders. The results show that the time correlation is the reason for the strong persistence of the weekly trading volume, but that time correlation also affects the scaling effect of *q*. We suspect that, in the Chinese stock market, this may be because weekly trading volume is affected by holidays.

### 4.2. The Complexity Synchronization Characteristic of Orders

**Figure 8:**
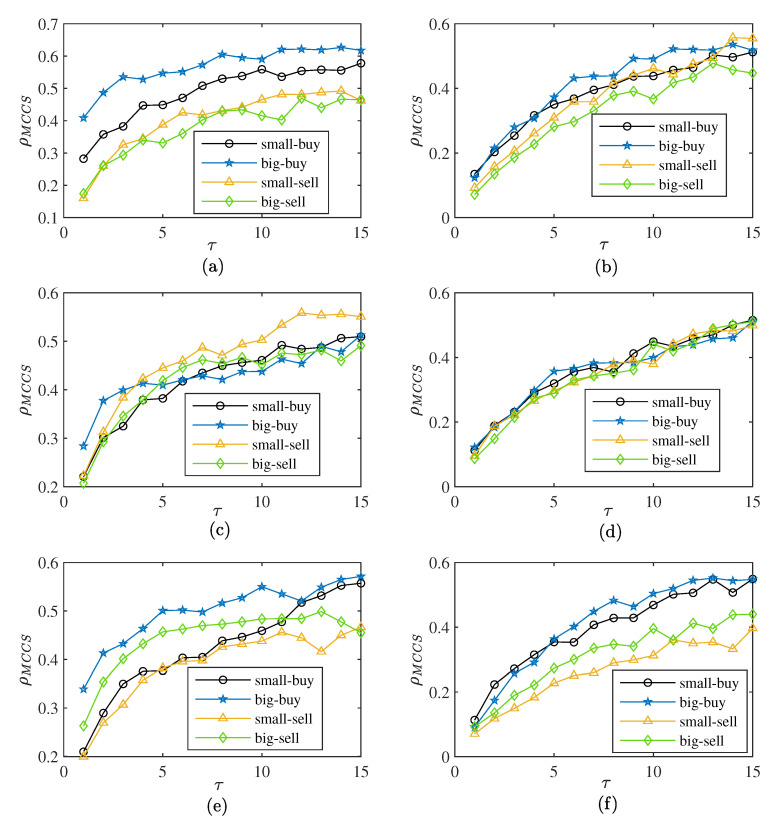
The complexity synchronization of buying and selling-out transactions, MCCS correlation coefficients with p=2 of (**a**) original sequence of CSI300, (**b**) shuffled sequence of CSI300, (**c**) original sequence of SSE50, (**d**) shuffled sequence of SSE50, (**e**) original sequence of CSI500, and (**f**) shuffled sequence of CSI500.

To determine whether big or small orders force the transaction, we calculated the synchronization of the different orders and total volume. Figure 7 shows the situation for the three markets when p=2. Figure 8a shows the obvious hierarchical characteristics of transactions in the CSI300 market. The synchronization of big order buying transactions is higher and the synchronization of big order selling-out transactions is lower. The synchronization difference in buying transactions is greater than that of selling-out transactions in the CSI300 market. Figure 8c shows that there is no significant difference in the synchronization of the SSE50 market. When τ is less than four, the big order buying transaction is higher, while, when τ is greater than four, the synchronization of small order selling-out transactions is more prominent. Figure 8e shows that the CSI500 market is similar to the CSI300 market. The buying transactions for big orders have the highest degree of synchronization, but for sell-out transactions, the synchronization of big orders is higher. Figure 8b,d,f is the complexity synchronization after shuffle according to the shuffle rules [25]. The synchronization of the three markets has different degrees of reduction, with the decline in the CSI300 market being the most obvious. This suggests that the synchronization results partly from the correlation of the time series. The stratification of the three markets also changes after the shuffle. The stratification phenomenon is not obvious after the shuffle in the CSI300 market. The synchronization of small order selling-out behavior is higher than that for big orders and small orders are the dominant force in selling transactions. The CSI500 market shows a more obvious stratification phenomenon after the shuffle. This suggests that the synchronization is weakened by the correlation of the time series in the CSI500 market. The synchronization of big orders is higher than that of small orders and big orders are the leading force in market transactions. For the relatively mature SSE50 market, there is no obvious dominant force in market transactions.

**Figure 9:**
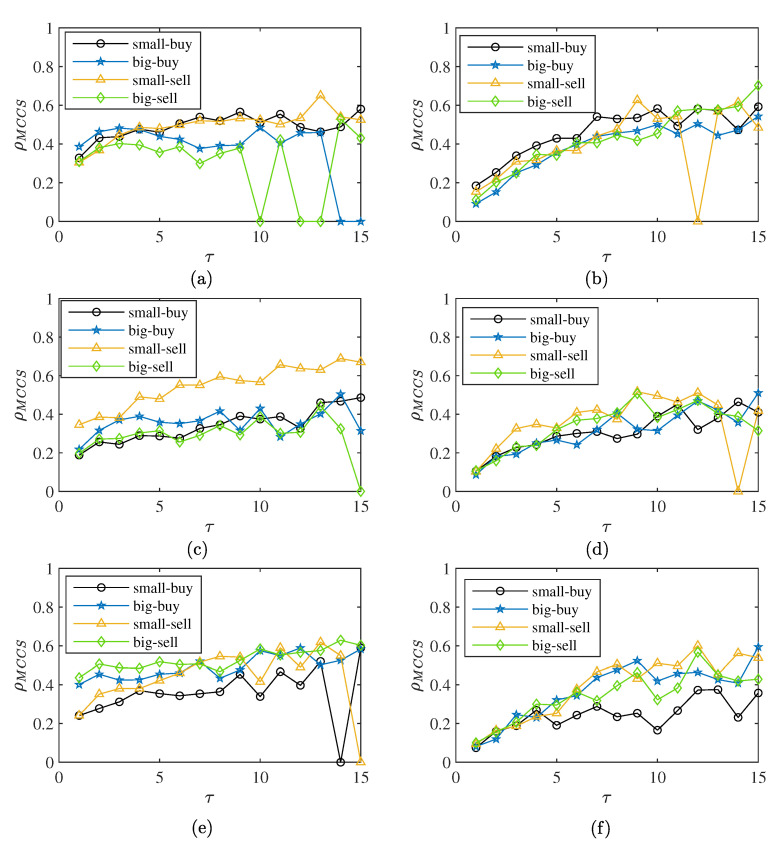
The complexity synchronization of buying and selling-out transactions with weekly volume, MCCS correlation coefficients with p=2 of (**a**) original sequence of CSI300, (**b**) shuffled sequence of CSI300, (**c**) original sequence of SSE50, (**d**) shuffled sequence of SSE50, (**e**) original sequence of CSI500, and (**f**) shuffled sequence of CSI500.

We present the complexity synchronization characteristic for weekly volume in Figure 9a. In Figure 9, small selling orders are shown to have stronger synchronization than big orders, which is consistent with the daily volume result, though this result is cut by time correlation. In Figure 9f, buy transactions are driven by big orders in the CSI500 market. In Figure 9d, there is no obvious driving force for trading in the SSE50 market, which is also consistent with the daily volume result.

**Figure 10:**
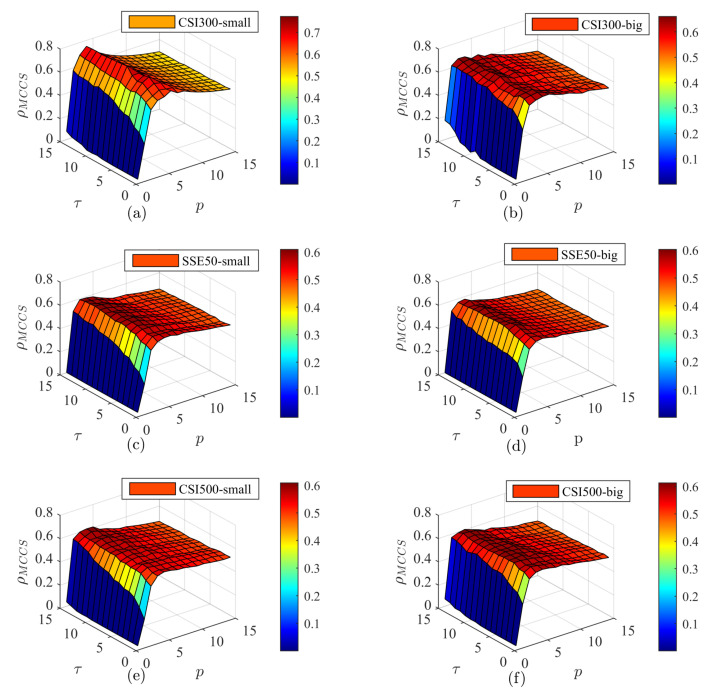
Three-dimensional diagram of the complexity synchronization of order-buying transactions. (**a**,**c**,**e**) is the small order behavior, (**b**,**d**,**f**) is the graph of big order behavior.

**Figure 11:**
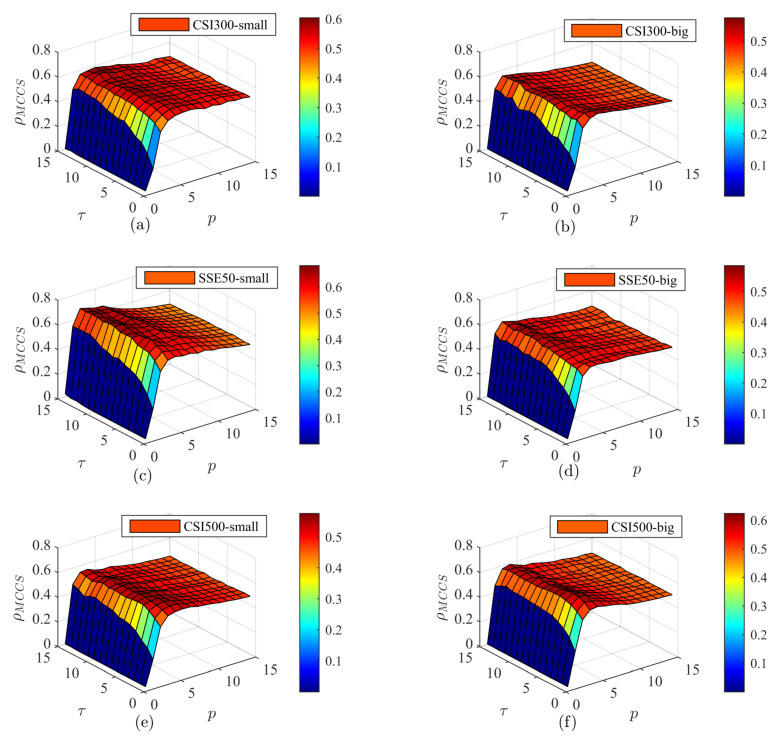
Three-dimensional diagram of the complexity synchronization of order selling-out transactions. (**a**,**c**,**e**) is the small order behavior, (**b**,**d**,**f**) is the graph of big order behavior.

To further understand the complexity synchronization of the total volume and different orders in the three markets, three-dimensional diagrams are presented in Figure 10 and Figure 11 with a multiscale exponent *p* and a coarse grain factor τ. Figure 10 shows the buying transaction and Figure 11 shows the selling transaction. For buying and selling orders in different markets, with a change in *p*, the figures show the characteristics of first rapidly increasing and then remaining basically unchanged. With a change in τ, the figures show a positive correlation trend of different degrees. However, there are differences between the changes for the different transactions. In Figure 8, the Euclidean distance is used to measure the complexity invariant distance, where p=2. In Figure 10 and Figure 11, we use the Minkowski distance to measure the complexity invariant distance. Therefore, Figure 8 can be seen as a slice of Figure 10 and Figure 11 at p=2. For the variation in MCCS values, p=2 is in the increasing part and is a top plane at p>5. As τ increases, the synchronization gradually increases, especially when τ is less than five. This positive relationship is more obvious in small order buying transactions of the CSI500 market. Compared with Figure 10, the top plane of each plot in Figure 11 fluctuates to a greater extent. The fluctuation in Figure 10b is the most pronounced, exhibiting a rugged top plane and the top surface of the other plots is smoother. In the increasing part of the three-dimensional figure, as *p* increases, as shown in Figure 10b,f increases more rapidly, exhibiting a rapid color change in the figure. Figure 10a shows a noticeable drop when *p* is large, but the change is still smooth, showing a color change in the top plane. This implies that there is a significant peak in the MCCS value when p<5.

### 4.3. Discussion

The results of the fractal analysis indicate that the trading volume of the Chinese stock market shows strong persistence, which is caused by the correlation of time. When we remove the time correlation by shuffle, it is found that the persistence of small orders is more potent than that of big orders and that the Chinese stock market shows persistence of small fluctuations and anti-persistence of big fluctuations. We observed that selling trades are driven by small order transactions which are unaffected by the time correlation. However, there is no apparent driving force for buying trades. In the CSI500 market, selling transactions are driven by big orders. We think that this may be because the majority of retail investors are in the CSI500 market. Transactions are mainly carried out in small orders, but big orders, which have more considerable weight, are the dominant force in the CSI500 market.

Previous studies of the stock market microstructure have mostly focused on the relationship between technical indicators, including volume and price, and volume and volatility. These studies found interaction between variables. Studies of the microstructure of volume also focus on the temporal correlation of volume and the driving force of this correlation. We directly studied the complex behavior of volume itself to determine the driving force of volume. Early studies showed that volume drives volatility [7] and stock price [5], and some studies have shown that volatility drives volume [51]. For trading volume, institutional investors are a stronger driving force for volume correlation than individual investors [18] and trading volume itself has a strong time correlation [56]. Buying transactions are more inter-related than selling transactions [19]. Financial rules and shocks can also reduce trading volume [53,54]. We did not only consider the relationship between variables, but also focused on the variable itself. We used the MFDFA and MCCS methods, which utilize more nuanced market information than the econometric regression models used in previous studies. Earlier studies were based on microstructure analysis from the perspective of the relationship between variables, whereas, we explain the microstructure of the volume indicator itself.

## 5. Conclusions

We used MFDFA and MCCS methods to study the complexity behavior of small and big orders in the Chinese stock market and found that the driving forces of different markets were different. Our results are informative for market participants. Market investors can better understand the buying and selling transactions in the stock market, discern the intentions of different trading forces in the market, and gain more information from the trading volume in the investment. For emerging markets, such as the Chinese market, the results of CSI300 are more informative, and for developed markets, the results of SSE50 can be used as a reference. For policy makers, different policies need to be formulated for different markets due to the different drivers of different markets. For the SSE50 market, policies can be relatively lenient, while for the CSI500 market, which is driven by big orders, targeted policies can help improve market efficiency. Our study has certain limitations. We have only considered the absolute value of the volume, and have not undertaken in-depth investigation of the volatility of different orders—future studies may pursue this. In addition, our results may have been influenced by the global health crisis and it is sensible to recognize that this period differs from those without such crisis. Finally, the complexity analysis method can be applied to the study of other financial markets. This may include complexity synchronization between different futures markets and the complexity behavior of futures and stock markets.

## Figures and Tables

**Table 1:** Summary of previous literature.

Study	Market	Microstructure Variable	Method	Main Findings
Koubaa and Slim [7]	Developed and emerging stock market	Volume and volatility	Non-linear STFIGARCH model	Large volume drives the high volatility regime
Barclay and Hendershott [5]	US stock market	Volume and open price	Econometric model	Pre-open trading contributes to the efficiency of the opening price
Sheng et al. [8]	Major stock markets	Volume and return	AR-GARCH model	International return spillover effects are sensitive to different levels of trading activity
Suominen [52]	/	Volume, volatility and price	Game equilibrium model	Explanation of trading volume containing useful information for predicting volatility
Ormos and Timotity [54]	Budapest Stock Exchange	Investor trading	Probabilistic model	Evidence of changes in investors’ trading in the financial crisis
Wang et al. [53]	Chinese stock market	Price, volatility and volume	Econometric model	The circuit-breakers do not affect bid-ask spreads and reduce volume and trades
Louhichi [51]	CAC40 Index stock market	Volume and volatility	GARCH model	Supporting evidence for strategic asymmetric information hypothesis
Xu [55]	Chinese stock market	Volatility and volume	VAR model	High volatility is explained by its lagged volatilities and trading volume
Covrig and Ng [18]	US stock market	Volume	Dynamic regressive model	Institutional trading generates a more pronounced effect on volume autocorrelation than individual investor trading
Alvarez-Ramírez and Rodríguez [56]	US stock market	Volume	Detrended fluctuation analysis	The strength of correlations exhibits important temporal variations
Lee et al. [19]	Korean stock market	Volume	Correlation function	The properties of the correlations of buy and sell volumes differ
This study	Chinese stock market	Volume	MFDFA and MCCS	

**Table 2:** Descriptive statistics on the volume of the three markets.

Panel A: CSI300	Buy-Small	Buy-Big	Sell-Small	Sell-Big
Obs	1069	1069	1069	1069
Mean	$1.15\times 10^{7}$	$8.79\times 10^{7}$	$1.03\times 10^{7}$	$8.85\times 10^{7}$
Max	$9.96\times 10^{7}$	$6.09\times 10^{8}$	$4.50\times 10^{7}$	$5.29\times 10^{8}$
Min	$1.69\times 10^{6}$	$9.65\times 10^{6}$	$1.92\times 10^{6}$	$6.04\times 10^{6}$
S.D.	$8.81\times 10^{6}$	$6.24\times 10^{7}$	$6.16\times 10^{6}$	$6.34\times 10^{7}$
Skewness	3.27	2.00	2.05	1.86
Kurtosis	17.66	7.36	6.02	5.69
Jarque–Bera	15,802.23 ***	3123.95 ***	2364.75 ***	2055.29 ***
**Panel B: SSE50**	buy-small	buy-big	sell-small	sell-big
Obs	1069	1069	1069	1069
Mean	$2.03\times 10^{7}$	$1.02\times 10^{8}$	$1.80\times 10^{7}$	$9.66\times 10^{7}$
Max	$1.46\times 10^{8}$	$8.49\times 10^{8}$	$9.01\times 10^{7}$	$5.38\times 10^{8}$
Min	$3.62\times 10^{6}$	$4.34\times 10^{6}$	$4.31\times 10^{6}$	$1.01\times 10^{7}$
S.D.	$1.48\times 10^{7}$	$7.42\times 10^{7}$	$1.07\times 10^{7}$	$6.44\times 10^{7}$
Skewness	2.60	2.85	2.15	2.04
Kurtosis	10.58	15.51	7.30	6.71
Jarque–Bera	6187.74 ***	12,162.79 ***	3192.14 ***	2745.94 ***
**Panel C: CSI500**	buy-small	buy-big	sell-small	sell-big
Obs	1069	1069	1069	1069
Mean	$6.00\times 10^{6}$	$4.61\times 10^{7}$	$5.18\times 10^{6}$	$4.08\times 10^{7}$
Max	$4.02\times 10^{7}$	$2.83\times 10^{8}$	$2.18\times 10^{7}$	$3.40\times 10^{8}$
Min	$6.35\times 10^{5}$	$1.64\times 10^{6}$	$5.92\times 10^{5}$	$1.37\times 10^{6}$
S.D.	$4.33\times 10^{6}$	$3.38\times 10^{7}$	$2.94\times 10^{6}$	$3.35\times 10^{7}$
Skewness	2.52	1.80	1.42	2.33
Kurtosis	10.73	5.24	3.66	9.90
Jarque–Bera	6256.93 ***	1800.07 ***	955.60 ***	5333.14 ***

*** is significant at the 1% level.

## Data Availability

Publicly available datasets were analyzed in this study. This data can be found here: https://lareine66.github.io/complexity-behavior-data/.

## Abbreviations

The following abbreviations are used in this manuscript:

CSI300	China Securities 300 Index.
SSE50	Shanghai Stock Exchange 50 Index
CSI500	China Securities 500 Index
